# Traditional *diyabath* (fermented cooked rice) as a pre-breakfast meal versus none (breakfast only) in improving gut microbiome and nutritional, health and immune parameters of young women in Sri Lanka: study protocol for a controlled trial

**DOI:** 10.1186/s12906-026-05301-w

**Published:** 2026-02-24

**Authors:** AHGS Udari, Carukshi Arambepola, Tharanga Thoradeniya, Sharmila Jayasena, RHSK de Silva, Darshi Thoradeniya, Vithanage Pujitha Wickramasinghe

**Affiliations:** 1 Department of Biochemistry & Molecular Biology, Faculty of Medicine, University of Colombo, 25, Kynsey Road, Colombo, 00800 Sri Lanka; 2 Department of Community Medicine, Faculty of Medicine, University of Colombo, Colombo, 00800 Sri Lanka; 3 Faculty of Indigenous Medicine, University of Colombo, Rajagiriya, Sri Lanka; 4 Department of History, Faculty of Arts, University of Colombo, Colombo, 00800 Sri Lanka; 5 Department of Paediatrics, Faculty of Medicine, University of Colombo, Colombo, 00800 Sri Lanka

**Keywords:** Fermented cooked rice, *Diyabath*, Health status, Gut microbiome, Women of reproductive age

## Abstract

**Background:**

In developing countries, a significant number of women of reproductive age fail to meet their required intake of essential micronutrients through diet alone. Although nutrient supplementation is a common approach, it may not offer a sustainable solution, particularly in Asian countries like Sri Lanka, where healthcare is provided free from womb to tomb. Therefore, it is timely to explore nutritious and affordable local food options to enhance nutrition. *Diyabath*, a traditional meal prepared from overnight fermentation of cooked rice, is often used as a dietary therapy by indigenous medical practitioners in Sri Lanka. While the benefits of fermented foods in improving nutritional status and gut microbiome across all ages are well-known, the specific advantages of fermented cooked rice, such as *diyabath*, remain largely unexplored. This trial aims to evaluate the effects of *diyabath* on the gut microbiome, as well as on nutritional, health and immune parameters among young women of reproductive age in Sri Lanka.

**Methods:**

This is a single arm, non-randomized controlled trial among 45 women aged 20–35 years, purposively selected from a residential hostel in Colombo District, Sri Lanka. The primary outcomes: gut microbiome composition, nutritional status, and health and immune markers will be assessed before and after a 10-week control phase, during which participants will follow their usual dietary habits (control). In the subsequent 10-week intervention phase, the same participants will consume *diyabath* daily as a pre-breakfast meal (intervention). The *diyabath* will be prepared according to an optimal recipe identified through a comprehensive evaluation of traditional recipes during the intervention development phase. Outcomes will be reassessed immediately and one month after completing the intervention. Changes in outcomes between the control and intervention phases will be compared.

**Discussion:**

This study intends to evaluate the impact of introducing *diyabath* as a pre-breakfast meal on changes in host gut microbiome, nutritional status, and health and immune markers in young women in reproductive age. The findings may support the promotion of affordable indigenous dietary solutions, particularly in low-resourced rice-growing countries, to mitigate nutritional deficiencies and provide a basis for future research on traditional food.

**Trial registration:**

This protocol is registered in the Sri Lanka Clinical Trials Registry (SLCTR). Clinical trial number: SLCTR/2024/032 (Registration date: 09/10/2024).

## Background

Sri Lanka, a county in South Asia, has a wide variety of traditional rice and rice-based meals that link people with their rich culinary culture. This culture promotes sustainable food consumption practices, including a variety of unique preservation methods for maximum food utilization. *Diyabath* is one such traditional Sri Lankan pre-breakfast meal prepared by fermenting leftover cooked rice (from dinner) overnight, contributing to reduced food wastage. Diverse communities in Sri Lanka enhance the gastronomic value of *diyabath* by adding different ingredients such as chilies, onions and coconut milk at different stages of fermentation. This flavour infusion is a practice that not only improves the sensory quality but also adds nutritional and medicinal value. Therefore, there are different recipes of *diyabath* consumed by Sri Lankans since many decades. *Diyabath* is also used by Sri Lankan indigenous medical practitioners as a dietary remedy for conditions like gastritis and anaemia [1, 2].

In recent years, the consumption of fermented foods containing live microorganisms has emerged as an important dietary strategy for improving human health [3]. Fermentation of cereals and grains is shown to reduce the phytate activity leading to better absorption of vital minerals such as iron and zinc in the gut, and thereby enhance the bioavailability of protein [3, 4]. Further, it could produce certain desirable micronutrients such as vitamin B_12_ [5, 6]. In terms of health benefits, studies have shown positive outcomes upon consumption of fermented food in apparently healthy adults. A randomized interventional study reported enhancement of the gut health and immune parameters following a 17-week high-fermented-food diet [7], while another showed enhanced glucose metabolism following the consumption of sourdough bread (an Egyptian fermented food) [8].

A review on rice-based fermented foods showed that rice fermentation appears to be a potent tool in filling the nutritional gaps and enhancing global health. Rice fermentation is driven by the endogenous microorganisms as a natural fermentation, except for the rice-based alcoholic beverages such as *sake*. Fermentation of rice is primarily lead by lactic acid bacteria (LAB) such as *Lactobacillus bulgaricus*, *L. casei, Pediococcus acidilactici, Streptococcus faecalis, S. thermophilus* and *Saccharomyces* species, which help restore healthy intestinal flora [9]. Studies confirm that many of these LAB strains are probiotic candidates. Further, LAB produce antioxidants including active phenolic metabolites in fermented food. This blend of antioxidants and probiotics in rice-based fermented foods reveals their health benefits as functional food. Moreover, rice fermentation also yields compounds such as organic acids and amino acids including essential ones [10]. Germinated brown rice fermentation has shown anti-inflammatory properties due to the increase in its composition of bioactive compounds [11]. *Idli* is a traditional Indian food prepared from the fermentation of rice and black gram (lentil) as its main ingredients. The microbiome analysis of *idli* has revealed that the endogenous microorganisms present in *idli* produce short-chain fatty acids such as butanoate and propanoate. These fatty acids aid in lowering the pH and thereby enhance the bioavailability of minerals as well as inhibiting the harmful bacteria in the gut [12]. Thilagavathi et al. (2019) showed that the fermented cooked rice water is a good source of natural compounds with antimicrobial, antioxidant and anticancer activities. In addition to that, via an in-vitro assay, they claim that it effectively hinders the proliferation of hepatocellular carcinoma cells [13]. Further, *basi*, a fermented cooked rice preparation which is consumed as breakfast since many decades by ethnic people of Madhya Pradesh of India is also considered as a highly nutritious food. This is prepared by natural fermentation of cooked rice by soaking the cooked rice in water in an earthenware pot, hence very similar to *diyabath* [14]. According to Tiwari *et* al. (2019), the consumption of *basi* provides sufficient amount of essential nutrients such as iron, zinc, copper, manganese and potassium that may help cure blood disorder like anemia, improves malnutrition, enhance bone growth and muscle formation and reduces the chances of occurrence of diarrhea [14]. Therefore, *diyabath* can also be considered as a potential food to enhance health.

A Sri Lankan study reports that a *diyabath*-based spread contains good amounts of protein (5.2 ± 0.08%), polyunsaturated fatty acids (i.e. Linoleic acid: 9.74%) and micronutrients such as Vitamin B_12_ (2.3 µg/100 g)_,_ Iron (64.25 mg/100 g), Zinc (9.74 mg/100 g), Mg (167.79 mg/100 g) and Ca (184.94 mg/100 g) [15]. Two Sri Lankan reviews claim that *diyabath* is considered as a remedy for gastritis and also to reduce blood glucose levels due to its low glycaemic index [16, 17]. Further, according to Jayawardena and Wansapala (2015), *diyabath* contained *Lactobacillus* group bacteria at the end of 12 h fermentation period [15].

**Fig. 1:**
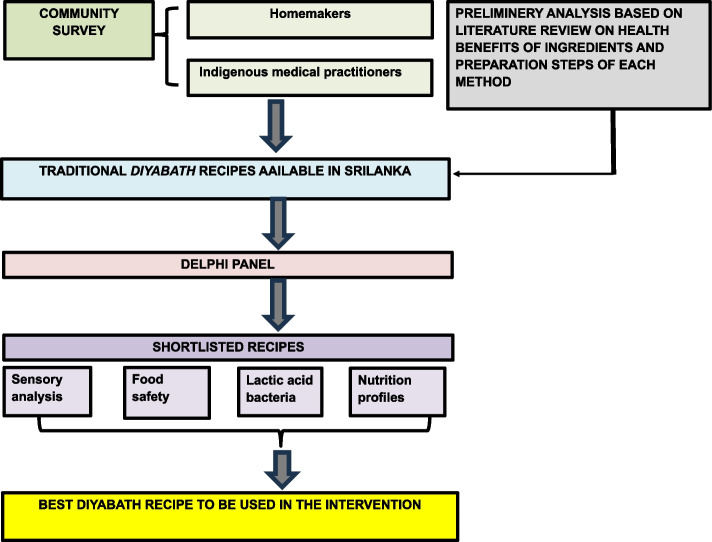
Flow of the intervention development

To achieve a holistic understanding of this traditional *diyabath* preparation in Sri Lanka, a multi-pronged approach was implemented (Fig. 1). Examination of traditional medicine scripts and the Ayurvedic pharmacopeia was followed by conducting archival scoping review to explore historical insight. Further, a systematic literature review was conducted to integrate current scientific knowledge on the functional and health benefits of fermented rice. A community survey was also conducted to gather traditional knowledge on *diyabath* preparation methods [18]. Potential participants for the survey were recruited purposively from all administrative provinces in Sri Lanka. The identified recipes were shortlisted via a panel discussion according to the modified Delphi technique [19]. The three shortlisted recipes were then prepared according to the developed standard operational procedures (SOP) and evaluated in view of its nutritional composition, microbiological and chemical safety and palatability. The results of the microbiological assays were compared with the recommended limits in the SLSI and regional standards such as FASSAI (Indian standard) and confirmed the microbial safety of *diyabath* [20–22]. Further, mineral analysis didn’t detect heavy metals in *diyabath*, therefore, is safe for the consumption.

According to our preliminary analysis, *diyabath* is rich in iron, magnesium, manganese and copper (with up to 15%, 8.78%, 19.6% and 31.1% of contribution respectively to the daily recommended value of each element per one serving of diyabath; one cup-230 g). The antioxidant activity in *diyabath* is high and comparable to other antioxidant-rich foods. The LAB count is in the range of 10^6^ CFU/g, making it a potential source of probiotic. Furthermore, the addition of red onions, roasted red chili and coconut milk for flavour infusion can also exert a prebiotic action as proven in previous studies [4, 9, 23–25]. Therefore, collectively, *diyabath* can serve as a beneficial ‘symbiosis’ that will enrich the gut microbiome. *Diyabath* made from different rice varieties were also tested for their sensory properties according to ISO 13299 [26]. Since *diyabath* made from parboiled *nadu* rice (Sri Lankan rice variety Bg 352) scored more for taste, it was selected for further investigations, thus prepare the intervention. Parboiling is a traditional post-harvest processing method of rice practiced in South Asia which consists of steeping the paddy in hot water, continuing to steam and then drying the steamed rice down to an appropriate moisture [27]. A Sri Lankan randomized cross over study using healthy volunteers concluded that the glycaemic index of *nadu* (Bg 352) is low (40 ± 4). Therefore, it has been recommended that, parboiled *nadu* is of nutritional significance to the individuals who are concerned on controlling their energy intake and glycaemic response [28]. The above results from our preliminary study and the closely related other studies assure that *diyabath* can improve gut microbiome as well as nutritional, health and immune parameters in human. However, there is limited published literature of scientifically evaluated data on the nutritional, immune and health impact of consuming *diyabath* or similar fermented rice-based foods, which will be accomplished via this clinical trial.

Micronutrient deficiencies, especially anaemia, are common among Asian women of reproductive age [29]. The World Health Organization (WHO) reports that 48% of pregnant women in such countries are anaemic [30]. Although most Sri Lankan pregnant women meet their recommended energy intake, they often fall short of essential micronutrients such as iron, zinc, folic acid and vitamins D, A and B_12_ through their diet [31, 32]. Recently, 29.1% of first trimester pregnant women were found to be anaemic, highlighting the inadequate pre-pregnant serum iron status of women in Sri Lanka [33]. This issue has been aggravated by the COVID-19 pandemic and economic downturns, leading to disrupted food supplies and poor affordability. Although nutrition supplementation programmes that target pregnant women are available in Sri Lanka, they may not be sustainable in a country that provides free healthcare from womb to tomb. In this context, evaluating low-cost, domestically prepared, yet nutritious food is a viable option to improve the nutrition of reproductive women in Asia. In particular, this intervention has the potential to benefit young women, who represent the primary age group for first-time pregnancies in Sri Lanka [34]. By improving their nutritional status and gut health, they may be better prepared to minimize complications during pregnancy [35]. Such an approach aligns well with the Sustainable Development Goals (SGD).

Studying the long-term benefits of *diyabath* could prove useful. As a dietary solution based on rice, which is the staple in most Asian countries, *diyabath* could economically address women’s nutrition, and thereby maternal and child health more effectively than food fortification or supplementation. It would be a feasible option as it can be prepared by overnight fermentation of leftover cooked rice without additional cooking, making it convenient for people with busy lifestyles. It is also a plausible solution to minimise food waste in Sri Lanka, where half of the biodegradable municipal solid waste is due to food waste [36, 37]. The findings of this study could also be adopted by other Asian countries with similar socio-cultural backgrounds and rice production; and contribute positively to ‘sustainable consumption’ in response to current food crises in the world.

The study aims to evaluate the changes in host gut microbiome composition upon *diyabath,* a traditional rice-based fermented pre-breakfast meal vs none (only the regular breakfast) in relation to host nutritional status, health biomarkers and immune parameters of young women of reproductive age in Sri Lanka.

## Methods

### Study design

**Fig. 2:**
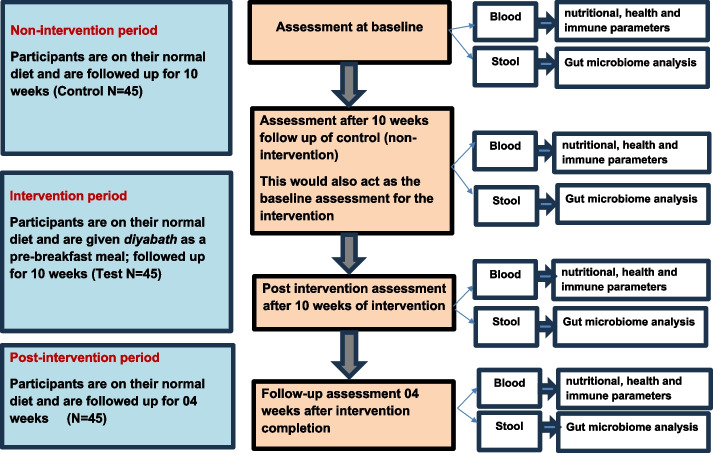
Flow diagram of the trial

This protocol is for a single arm, non-randomized, controlled trial (Fig. 2) reported in accordance with the SPIRIT statement. This design will ensure that factors, such as gut flora, digestion and other physiological variables, which could influence the study results, remain consistent in participants throughout the study period, as the same group of participants will serve as both the control and test groups. This consistency will enable reasonable comparisons with minimum effect due to individual differences. A wash-out period is not considered in the design, as *diyabath* is introduced as the intervention only after the initial control phase.

### Study setting

Participants for the study will be recruited from a women’s hostel located in Colombo District. This area is popular among young adults engaged in education and employment due to its proximity to several universities and workplaces within a 5 km radius. The hostel residents, women aged 20–35 years who have moved to this area for work or study, consume all three meals prepared daily in the hostel. By selecting this setting, it ensures consistent food intake across all participants and represents the population of apparently healthy young women of reproductive age in Sri Lanka.

### Study population

Women aged 20–35 years who have been residing in Colombo District for a minimum period of two years and with normal body mass index (BMI) (18.5 to 24.9kg/m^2^) will be recruited.

Pregnant and lactating women; those on long term medication for metabolic illnesses, severe concurrent infections, bowel disorders or intolerance to cereal based foods, coconut milk, red onion and dried chili (diagnosed following a clinical assessment made by a medical practitioner along with perusal of diagnosis cards, clinic records and treatment prescriptions for confirmation); women already consuming *diyabath* or any nutritional supplementation; or on antibiotics at the time of the study or within past two weeks will be excluded from the study.

### Intervention

The intervention consisted of a pre-determined portion of *diyabath* (1 cup or 230 g) measured and served in standardized containers, on every alternate day (week days only) during the 10 weeks of intervention period.

### Outcomes

**Fig. 3:**
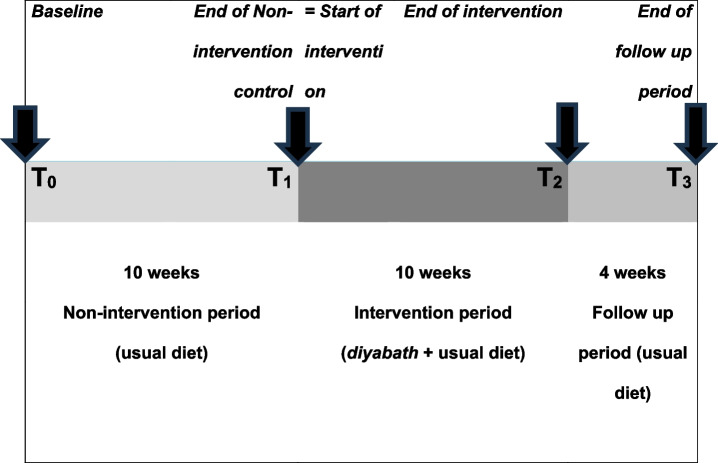
Time structure of the trial

Outcomes will be measured at four time points: at the baseline (T_0_), after completion of the 10-week non-intervention control period (T_1_), after completion of the 10-week intervention period (T_2_) and 4 weeks after completion of the intervention (T_3_) (Fig. 3).

Primary outcomes of the study include changes in the following parameters:

- *Host gut microbiome composition:* Evaluated using percentage abundance of probiotic strains (i.e. *Lactobacillus* spp, *Bifidobacterium* spp) and other beneficial microorganisms (i.e. *Oscilllospira*, *Akkermansia muciniphila*), alpha-diversity index and firmicutes-to-bacteroidetes ratio (F/B). Diversity index reveals the change in the gut microbiome composition and is important to have a balanced ratio between the most common two phyla (Firmicutes and Bacteroidetes F/B) for homeostasis of the gut [38]. Gut microbiota is directly responsible to develop digestive, immunological and metabolic functions in host [39, 40].
- *Nutritional status and health parameters*: Evaluated by serum micronutrients and serum ferritin
- *Health biomarkers*: Assessed by lipid profile, high-sensitivity C-reactive protein (hsCRP), fasting blood sugar (FBS), aspartate aminotransferase (AST), alanine aminotransferase (ALT) and serum insulin
- *Immune markers:* Evaluated by analysing interleukin-6 (IL-6), IL-10 and cytokines

Each participant is expected to improve these parameters to either reach a healthy limit or a healthier end if already in the healthy limit.

Secondary outcomes of the study include:

- *Antioxidant capacity:* An increase in total antioxidant capacity in serum after consumption of an antioxidant rich food indicates absorption of antioxidants from the food and an enhanced in vivo defence status against oxidative stress [41].
- *Anthropometry:* Measured as waist circumference, hip circumference and BMI

### Sample size

The study is designed to detect a significant difference in the pre- and post-intervention time points for a given dichotomous variable [42, 43].

$$\mathrm N=\left\{{\mathrm p}_1\;\left(100-{\mathrm p}_1\right)+{\mathrm p}_2\;\left(100-{\mathrm p}_2\right)\right\}\times{\mathrm f}_{\left(\mathrm\alpha,\mathrm\beta\right)}/\left({\mathrm p}_1-{\mathrm p}_2\right)^2$$

Where;

$$\mathrm{N}=\text{Minimum sample size required for each group}$$

$$\begin{aligned} \alpha=\:&\text{The level of significance used for detecting} \\& \text{an intervention difference}\:(0.05) \end{aligned}$$

$$\begin{aligned} 1- \beta=\:&\text{The degree of certainty (power) that the difference p1-p2}, \\&\text{if present would be detected, often set at 0.8 (0.2)} \end{aligned}$$

$$f=\text{A function of}\ \alpha\ \mathrm{and}\ \beta\ \mathrm{when}\ \alpha\ \text{is 0.05 and}\ \beta\ \text{is 0.02 (7.9)}$$

$$\begin{aligned} \mathrm{P}_1=\:&\text{Expected percentage of an outcome variable}\\& \text{at the baseline of the intervention} \end{aligned}$$

$$\begin{aligned} \mathrm{P}_2=\:&\text{Expected percentage of an outcome variable} \\&\text{at the end of intervention} \end{aligned}$$

Previous interventional studies on fermented food have shown that host nutritional status, health and immune parameters are predominantly affected by the change in host gut microbiome [42–45]. Therefore, change of gut microbiome composition was used for sample size calculation. Previous studies have used the percentage presence of selected taxonomic groups of microbiota to interpret the gut microbiome changes [7, 46, 47]. Therefore, P1 and P2 in this study were derived based on a 17-week randomized controlled intervention study (*n* = 18, healthy adults) on high fiber food-based diet where percentage presence of *Oscillospira* at baseline was 31% and at the end of the intervention was 73% [7].

Therefore;

- P_1_ = Expected percentage presence of Oscillospira at T_1_ = 31% [7]
- P_2_ = Expected percentage presence of Oscillospira at T_2_ = 73% [7]

The sample size obtained (*n* = 18.41) was corrected to account for design effect due to clustering (d). In most interventional studies, the maximum d has been reported as 2, and therefore the design effect was considered as 2 for this trial [48, 49]. Therefore, after correction, the sample size was 36.82. With another 25% non-response added, the final sample size was 45 participants.

### Recruitment

The study setting has on average 80 women hostellers. The principle investigator (PI) will approach them through hostel authorities and will conduct an introductory session on the intervention procedure, potential risks and benefits of the study. Those who express interest will be given an information sheet in local languages (Sinhala and Tamil), with sufficient time to review the information and opportunities to ask questions and seek clarification from the investigation team. Using the hostel registry, potential candidates will then be identified using simple random sampling and screened for their eligibility for the study. This screening will include a clinical assessment including BMI performed by a medical officer who is not involved in the study. Relevant documents will be reviewed to confirm certain exclusion criteria. In addition, an interviewer-administered food frequency questionnaire (FFQ) validated for Sri Lankan adults will be administered to get a clear understanding of the participant’s usual dietary pattern [50]. Those already consuming *diyabath* or any other rice-based fermented food will be excluded from the study. Once eligibility is established based on the screening results, the potential candidates will be invited to participate in the study and informed written consent obtained by the PI.

### Implementation

**Fig. 4:**
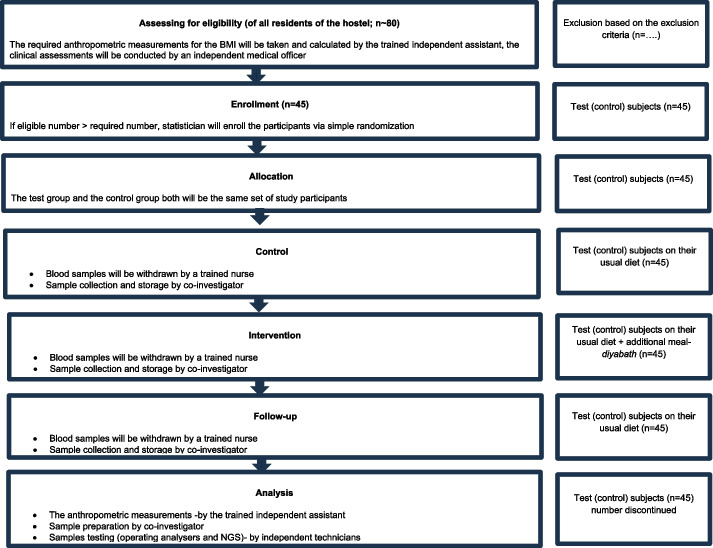
CONSORT flow chart for the trial

Since this is a single-arm trial, there is no randomization into groups; and the same group of participants will be part of both the control and intervention phases with no specific mechanism for assigning or sequence concealment (Fig. 4 and Table 1).

**Table 1:** Participant timeline

Task	Study period (weeks)													
	**Recruitment**	**Non-intervention control period**						**Intervention period**					**Post-intervention follow up**	
ENROLLMENT - Eligibility screening - Informed consent	−2	0^a^	2	4	6	8	10^b^	12	14	16	18	20^b^	21	22^b^
	X													
	X													
Maintaining a food diary (24 h dietary records)		x	x	x	x	x	x	x	x	x	x	x	x	x
Baseline control assessment		x												
Anthropometry (height, weight, Waist circumference, Hip circumference, BMI)		x					x					x		x
Collection of blood samples (fasting) - serum micronutrients & serum ferritin - serum lipid profile - serum free fatty acids - AST, ALT - hsCRP, IL-6, IL-10, Cytokines - FBS & serum insulin		x					x					x		x
Collection of stool samples - Gut microbiome analysis (% abundance, alpha-diversity index, F/B ratio) - Short chain fatty acid profile		x					x					x		x

^a^assessment is done at the beginning of the week

^b^assessment is done at the last day of the week

Participants will be informed not to take part in any other nutritional intervention as well as not consume any nutritional supplementations including other cooked rice-based fermented foods during the study. A list of such food items will be given to them for reference at the beginning of the study. In addition, each participant will be asked to record daily all the foods that they have eaten other than what is provided by the hostel throughout the intervention phase. These records will be checked by an investigator during weekdays. Set reminders will be sent daily via SMS to all participants to keep records of their diets and will be asked to acknowledge the receipt of the message.

During the intervention period, every morning, the participants will be reminded via SMS to be present at the breakfast table. Standard portions of *diyabath* will be placed in the dining area before breakfast, ensuring uniformity in presentation across all participants. One investigator will be present at the site to observe the participants, to ensure that they consume *diyabath* as intended and answer any queries. The participants are requested to finish the served amounts and return the containers. If there is any leftover *diyabath*, the investigator will weigh it and make a record of it. This procedure will be repeated daily for 10 weeks during the intervention phase.

#### Discontinuation of the intervention

Participants of the study can voluntarily withdraw their consent and participation at any time during the study. In addition some participants would be compelled to take antibiotics or nutritional supplements during this period. In such instances, those who took antibiotics for 5 days or more will be discontinued. The discontinuation of those who started taking any nutritional supplement during the course of intervention will be considered case by case. Further, based on the dietary data records, participants who consumed other fermented cooked rice- based food for more than 4 days during the study period will be also discontinued from the study. In addition to that, participants will be discontinued from the study if they report any adverse events that may produce lethal outcomes.

#### Compliance and drop-outs

Based on the total amount of *diyabath* consumed throughout the intervention, as per the records, participants whose compliance with the consumption of *diyabath* is ≤ 70% of the total dose will be considered as dropped-out.

To enhance compliance, the participants will be briefed on the importance of continuing with the study and the potential health benefits. They will be informed that they will receive their test results with regards to biochemistry and body anthropometry, which will be an incentive to remain in the study.

### Data collection and storage

At the specified time points, anthropometric measurements will be taken by a trained assistant and blood samples drawn by an experienced nurse. Stools samples will be collected by the investigators and the gut microbiome analysis by a laboratory technical officer. All the outcome assessors will be blinded to the participants’ status. Due to the nature of this study neither trail participants nor the investigators will be blinded. All measurements will be based on standard methods followed using calibrated equipment.

#### Anthropometry

To minimise variability, each instrument will undergo the required calibration process, and all measurements taken by the same person (trained assistant) using the same equipment, as per the International Society for Advancement of Kineanthropometry (ISAK) protocol [51]. Measurements will be taken in duplicate at the same premises. If the mean of the two values exceeds a pre-determined threshold, a third measurement will be taken. Height will be measured in duplicate to the nearest 0.1 cm using a stadiometer (Seca 213, Seca gmbh & co); weight to the nearest 0.1 kg using a calibrated electronic scale (Seca 763); and waist and hip circumferences using a non-stretchable measuring tape (Seca 200). Body mass index (BMI) will be calculated as weight in kg/height^2^ in meters.

#### Biochemical assessments

A venous blood sample of 10 ml will be drawn from each participant under aseptic conditions after an overnight fast (12 h). This will be done at the hostel premises, while half of the blood volume will be centrifuged within one hour, and the serum/plasma separated, aliquoted and kept frozen at − 20 °C until further analysis. The remaining blood will also be stored as whole blood. The samples will be analysed as given below at the Faculty of Medicine, University of Colombo, Sri Lanka.

- *Micronutrients:* Serum Folate, Vitamin D and serum ferritin using the MAGLUMI® chemiluminescence immunoassay (CLIA) and serum calcium level using commercially available kits
- *Health and immune biomarkers:* Serum lipid profile, FBS, AST, and ALT using commercially available kits, and hsCRP, serum insulin and immune markers (IL-6, IL-10) using the MAGLUMI® chemiluminescence immunoassay (CLIA).

Further, antioxidant capacity will be analysed using colorimetric ABTS method at the Industrial Technology Institute, Sri Lanka [52].

#### Stool sample collection and storage

Samples collected into stool specimen collection containers will be stored at −80℃ at the Faculty of Medicine, University of Colombo, Sri Lanka. The samples will be assessed for metagenomics based on bacterial 16S RNA (V3-V4 region) and fungal ITS1 region using illumina platform [7]. The analysis will be carried out at Credence Genomics (Pvt) Ltd., Sri Lanka by trained analysts.

The collected stool and blood samples will be subjected to bio banking, preserving at −80^0^C for future analysis.

### Statistical analysis

Data will be analysed using the statistical package for the for the Social Sciences (SPSS) version 21.0. The changes in nutritional, health and immune parameters between the baseline (T_0_) and at the end of control phase (T_1_) will be analysed first to identify any significant change with time. The end point of control phase will serve as the pre-intervention point for further assessments. The changes in outcomes between the pre-intervention point (T_1_) and at the end of intervention phase (T_2_) will be analysed to determine any significant effects of *diyabath* consumption on the study group.

Paired t-test will be performed to determine the significance of the mean difference of pre- and post- measurements during the intervention phase. *P* value less than 0.05 is considered statistically significant.

$$\begin{aligned} {\mathrm H}_0=\:&\left[\mathrm{Pre}-\mathrm{intervention}\;\mathrm{measurements} -\mathrm{post}\right. \left.-\:\mathrm{intervention}\;\mathrm{measurements}\right]=0 \end{aligned}$$

The same analysis will be performed in relation to the control phase. This evaluation is important to reveal any possible change in parameters that can occur due to seasonal, physiological or climatic changes in individuals.

Further, independent sample t-test will be performed to assess the significance between the intervention and control phases, in relation to the mean change in pre- and post-measurements observed during the intervention and control phases.

$$\begin{aligned} {\mathrm H}_0=\:&\left[\mathrm{Mean}\;\mathrm{change}\;\mathrm{in}\;\mathrm{control}\;\mathrm{phase} \right. \left. -\:\mathrm{Mean}\;\mathrm{change}\;\mathrm{in}\;\mathrm{intervention}\;\mathrm{phase}\right]=0 \end{aligned}$$

Bioinformatics analysis of metagenomics of microorganisms evaluated from stool samples will be carried out based on16S amplicon sequence variants (ASVs) model.

Analysis of the effect of diyabath on primary outcomes will be based on modified intention-to-treat (mITT) and per-protocol (PP) principles. The mITT population will include participants who completed the first 15 weeks of the study period which covers the control phase and half of the intervention phase. The PP population will include the participants who completely adhered to the study protocol [53]. Missing data will be adjusted with multiple imputation.

### Ethics and dissemination

This study has obtained ethical clearance from the Ethics Review Committee (ERC), Faculty of Medicine, University of Colombo, Sri Lanka (EC-23–121). Progress review will be conducted by ERC every six months until the trial is complete, where progress of the ongoing trial activities, any changes in the protocol and challenges will be reviewed and discussed. There is no specific trial steering committee allocated to this trial, other than the Data Monitoring Board (DMB). This board comprises two professors who are permanent staff members of the Faculty of Medicine, University of Colombo and with specialized areas of biochemistry and statistics. They are neither investigators of this project nor have any competing interests. The contact details of these members will be shared with the study participants requesting them to contact for any concerns. The DMB will independently review the queries and compile a report which will be reviewed along with the progress of the trial by the ethics review committee. Further, the amendments to the protocol will be immediately notified by the PI to the Sri Lankan Clinical Trials Registry in which the trial is registered; and if relevant, to the trial participants and regulators.

The food being tested in this intervention has been consumed by Sri Lankans for decades, and the preliminary survey has indicated no adverse effects*.* The food safety assessments carried out have already confirmed its compliance with reference safety values*.* Moreover, *diyabath* for the intervention will be produced according to an SOP, ensuring compliance with all relevant general hygienic practices. The total microbial counts, counts of pathogenic indicators and the heavy metal content will be tested in randomly collected samples of *diyabath*. Further, venipuncture will be carried out by an experienced nurse. Therefore, no adverse events are anticipated during implementation of the trial. However, participants will be provided with contact information of the investigators, an independent medical officer and DMC members to report any allergies, adverse events or changes experienced in the appetite, stool consistency, frequency and other concerns following consumption. The information sheet provided will also indicate the ingredients that are used in *diyabath* recipe (rice, coconut milk, red onion, roasted chilli, black pepper powder) so that those with allergies could refrain from participating in the study. Given these precautions, the intervention is expected to pose zero to minimal harm to participants.

The collected data will be stored as hard copies in a locked cupboard at the Faculty of Medicine, University of Colombo and in a password protected electronic database for at least 5 years. Confidentiality of the collected data will be assured. All the collected biological samples and data will be coded and analysed. The names of participants will not be used for any purpose other than to send their test reports. For any new analysis to be done using bio banking, ethics approval as well as re-consenting would be done.

The study findings will be disseminated in scientific publications regardless of the magnitude or direction of effect. If required, access to the participant level-data and statistical code will be decided upon on case-by-case basis, upon the recommendation of the ERC and DMB.

## Discussion

There is currently no scientific data available on traditional fermented rice-based pre-breakfast meals like *diyabath*, making it essential to gather traditional knowledge about it from community and indigenous medical practitioners and to scientifically evaluate its properties and functionality, in order to develop interventions that are of practical value in low-resource countries where rice constitutes the staple diet. This methodology could serve as a model to evaluate other indigenous food in the future.

One of the major challenges in interventions based on traditional food is maintaining the consistency in its composition and texture. To address this, the trial plans to strictly adhere to the developed SOP. However, since the fermentation can still vary depending on the environmental conditions such as temperature, especially when conducted domestically, therefore, *diyabath* for the intervention will be prepared in the same place throughout the study. Furthermore, to check whether microbial composition of *diyabath* drastically, samples of *diyabath* provided for the intervention will be analysed randomly during the study.

Previous studies have reported considerable variability over time in the patterns of gut microbiome diversity among individuals undergoing the same food-based intervention [7]. This variability may be due to differences in metabolic conditions, such as inflammation levels. Therefore, using same participants as the test and control groups will minimize the impact of factors such as food preferences, individual metabolism and other physiological variables on the comparisons between test and control groups, revealing the true impact of the intervention on each individual.

The percentage presence of *Oscillospira* at baseline vs at the end of the intervention was considered for the sample size calculation from a previous study which was conducted to evaluate the impact of fermented and high fiber food consumption on gut microbiome composition [7]. Of the reported organisms in the microbiome studies *Oscillospira* was identified as a genera which is constantly detected by 16S rRNA gene surveys of the human microbiome [46]. Furthermore, it has been identified that the abundance of *Oscillospira* is positively correlated with the diversity of gut microbiota [54]. It has been associated with improved markers of health and negative correlations with gut related diseases [54, 55]. Therefore, detection of the presence of *Oscillospira* is considered a reliable marker to evaluate the health of gut. The calculated sample size is compatible with previous studies that report significant changes between the pre-post consumption of fermented and other food on gut microbiota compositional, health, immune and nutritional parameter [43, 56–58].

Moreover, this design will include provisions to minimize the impact of consuming other fermented or similar foods. Such consumption by study participants will be closely monitored. In the information sheet, participants will be informed to refrain from taking fermented cereal foods as much as possible during the intervention period. The consumption of other types of fermented foods, such as yogurt will be reviewed through 24-h food re-call records. Furthermore, if any considerable deviation from the hostel menu is revealed, impact of these confounders will be analysed case by case using comparison of the results from the control period with those from the intervention period.

If successful outcomes are shown, this intervention programme will be promoted among the community and especially among young women of reproductive age. This will help highlight and popularize *diyabath,* which is currently less recognized as a low-cost, sustainable and nutritive solution for enhancing the health status of Sri Lankan women. The results are applicable to other Asian countries of similar socio-cultural backgrounds, where rice is the staple food.

### Trial status

Protocol version has been approved by the the Ethics Review Committee of the Faculty of Medicine, University of Colombo, Sri Lanka (Ref: EC-23–121-2.1.1). Further, this trial has been registered in the Sri Lanka Clinical Trials Registry (SLCTR): SLCTR/2024/032 (Registration date: 09/10/2024).

## Data Availability

No datasets were generated or analysed during the current study.

## Abbreviations

IMP: Indigenous medical practitioners
WHO: The world health organization
SOP: Standard operating procedures
SLSI: Sri Lankan standard institute
SLS: Sri Lankan standards
MRS: De Man–Rogosa–Sharpe agar
FASSAI: Food Safety and Standards Authority of India
FDA: Food and Drug Administration (US)
HACCP: Hazard analysis and critical control points
AOAC: Association of Official Analytical Chemists
HPLC: High-performance liquid chromatography
ICP-MS: Inductively coupled plasma mass spectrometry
GC: Gas chromatography
ITI: Industrial Technology Institute
SCFA: Short chain fatty acids
NGS: Next generation sequencing
BMI: Body mass index
SD: Standard deviation
FFQ: Food frequency questionnaire
HsCRP: High-sensitivity C-reactive protein
FBS: Fasting blood sugar
AST: Aspartate aminotransferase
ALT: Alanine transaminase
IL: Interleukin
PUFA: Poly unsaturated fatty acids
ISAK: International Society for the Advancement of Kinanthropometry
CLIA: Chemiluminescence immunoassay
ABTS: Azino-bis-(3-ethylbenzothiazoline-6-sulfonic) acid
ERC: Ethics review committee
DM: Data monitoring committee
CTRSL: Sri Lanka Clinical Trial Registry
